# Psychological resilience is correlated with dynamic changes in functional connectivity within the default mode network during a cognitive task

**DOI:** 10.1038/s41598-020-74283-7

**Published:** 2020-10-20

**Authors:** Takashi Miyagi, Naoya Oishi, Kei Kobayashi, Tsukasa Ueno, Sayaka Yoshimura, Toshiya Murai, Hironobu Fujiwara

**Affiliations:** 1 Department of Psychiatry, Graduate School of Medicine, Kyoto University, Kyoto, Japan; 2 Medical Innovation Center, Kyoto University Graduate School of Medicine, Kyoto, Japan; 3 Integrated Clinical Education Center, Kyoto University Hospital, Kyoto, Japan; 4 Department of Neurodevelopmental Psychiatry, Habilitation and Rehabilitation, Kyoto University, Kyoto, Japan; 5 Artificial Intelligence Ethics and Society Team, RIKEN Center for Advanced Intelligence Project, Tokyo, Japan

**Keywords:** Cognitive neuroscience, Stress and resilience, Human behaviour

## Abstract

Resilience is a dynamic process that enables organisms to cope with demanding environments. Resting-state functional MRI (fMRI) studies have demonstrated a negative correlation between resilience and functional connectivities (FCs) within the default mode network (DMN). Considering the on-demand recruitment process of resilience, dynamic changes in FCs during cognitive load increases may reflect essential aspects of resilience. We compared DMN FC changes in resting and task states and their association with resilience. Eighty-nine healthy volunteers completed the Connor–Davidson Resilience Scale (CD-RISC) and an fMRI with an auditory oddball task. The fMRI time series was divided into resting and task periods. We focused on FC changes between the latter half of the resting period and the former half of the task phase (switching), and between the former and latter half of the task phase (sustaining). FCs within the ventral DMN significantly increased during “switching” and decreased during “sustaining”. For FCs between the retrosplenial/posterior cingulate and the parahippocampal cortex, increased FC during switching was negatively correlated with CD-RISC scores. In individuals with higher resilience, ventral DMN connectivities were more stable and homeostatic in the face of cognitive demand. The dynamic profile of DMN FCs may represent a novel biomarker of resilience.

## Introduction

Human responses to stress vary widely. Some people develop stress-related disorders, such as posttraumatic stress disorder (PTSD) and depression; others develop mild to moderate psychological symptoms that resolve rapidly; and others report none of these symptoms in response to stress. Inter-individual variability in stress responses depends on many factors, including those that are genetic, developmental, cognitive, psychological, and neurobiological^1^.

Psychological resilience (hereafter referred to as resilience) is the process by which an organism adapts to an environment in the face of demanding and stressful stimuli, and can be thought of as “bouncing back” from a difficult experience^2^. Several brain regions are involved in resilience, such as the medial prefrontal cortex, anterior cingulate cortex, thalamus, hypothalamus, insula, amygdala, and hippocampus^3–6^. However, the way in which these multiple brain regions are recruited in the process of resilience (e.g., by networking, synchronising, or acting separately) is not clear.

Functional connectivity (FC) is defined as the temporal correlation between neuronal activity patterns of anatomically different brain regions^7,8^. The default mode network (DMN) is one such large-scale brain network. Raichle et al. used positron emission tomography to identify the baseline state of the human brain, termed the “default mode of brain function”^9^. Subsequently, resting-state FC analysis has provided evidence for the presence of a cohesive DMN^10^. The DMN is assumed to play an important role in the maintenance of baseline cognitive processes associated with self-awareness, autobiographical memory retrieval, anticipatory cognitive processes to plan for the future, the modulation of internal versus external tasks, and consciousness^11^. Spontaneous thoughts during a resting state, namely “mind-wandering”, may also modulate this network^12,13^. The core regions of the DMN consist of the ventral medial prefrontal cortex, posterior cingulate/retrosplenial cortices, inferior parietal lobule, lateral temporal cortex, dorsal medial prefrontal cortex, and hippocampal formation^11^. In addition, several studies suggest that the network comprises two functionally different subdivisions^14,15^, the dorsal DMN and the ventral DMN. The dorsal DMN, which involves the anterior and dorsal regions of the network, seems active when people make self-relevant decisions or engage in cognitive processes involving affect. The ventral DMN, which includes the medial and posterior temporal regions, acts during decision-making related to a scene based on semantic or episodic memory.

Regarding the relationship between resilience and FC, FCs within the DMN have been studied in relation to the effect of resilience on health outcomes^16^. FCs within the DMN have been found to be negatively correlated with resilience in healthy participants in the resting state^17^. One study showed that in PTSD patients (who generally have low resilience), FC in the ventral (not dorsal) DMN subsystem, which includes the medial temporal lobe (implicated in autobiographical memory processes), was lower than that in healthy participants^18^.

Although several studies have found possible relationships between resting-state FCs and resilience, to our knowledge, no study has investigated dynamic FC changes in the DMN during changes in cognitive state, specifically, during the period of transition from the resting state to a state of engagement in a cognitive task (“switching”) and during the course of the task (“sustaining”). FC is known to change dynamically over time^19^; one study reported that cognitive states could be correctly identified from covariance matrices estimated using as little as 30–60 s of data^20^. Resilience is conceptualised as a dynamic process; that is, it is thought to encompass positive adaptation to psychological stresses via various cognitive functions. Thus, studies examining the functional correlates of resilience that involve not only data collected during the resting state but also data collected during a task are important, because dynamic changes in DMN connectivity under conditions of increased cognitive load could capture processes intrinsic to resilience.

Of the various cognitive functions, attention is likely to be closely involved with resilience. One study showed that attentional control was positively related to resilience in healthy individuals^21^. Further, attentional dysfunction, particularly a decline in sustained attention, is associated with low resilience in individuals with PTSD^22^ and those with depression^23^. Stress or trauma-related tasks have been used in most fMRI studies examining the neural underpinnings of resilience^24^. However, the concept of resilience connotes cognitive aspects in addition to emotional ones, as reflected, for instance, in some items on one representative resilience questionnaire (the Connor–Davidson Resilience Scale: CD-RISC; e.g., Item 14, “Under pressure, focus and think clearly”). Thus, in this study, we focused on cognitive aspects of resilience, rather than emotional aspects. Additionally, to minimise behavioural variance, which is considered a major shortcoming of fMRI studies, we used a basic attentional task with relatively low cognitive load^25^. In the oddball paradigm, which we used in this study, participants are required to identify rare and unpredictable target stimuli presented among a stream of frequent non-target stimuli^26,27^.

In the current study, we used functional MRI (fMRI) and a relatively simple sustained attention task to investigate the association between resilience in healthy participants and changes in FC within the dorsal and ventral DMN during “switching” (from resting state to a task) and “sustaining” (during a task demanding cognitive effort).

We hypothesised that resilience would be correlated with the magnitude of FC changes within the DMN during “switching” and “sustaining”.

As noted above, we focus on the DMN, referring to studies of healthy individuals and patient studies of PTSD and depression. However, there may be other networks associated with resilience. Among them, we are interested in the salience network (SN) and the attention network (AN). Some regions within the SN are known to be associated with resilience^28,29^. Additionally, the SN has been identified as a switching network between rest and task^30^. The AN is a network that is activated by an attention task^27^. Therefore, we investigated the relationship between dynamic changes of FC and resilience not only for the DMN, but also for the SN and the AN to widely explore the neural basis of resilience.

## Results

### Sample characteristics

We recruited 92 participants. However, three participants were excluded because they made head movements during the fMRI. Finally, 89 participants were included in the analysis (Table 1). The mean age of participants (*n* = 89) was 32.1 years (range 18–68). The mean age of male participants (*n* = 59) was 34.2 years (range 20–66) and that of female participants (*n* = 30) was 27.8 years (range 18–68).

**Table 1:** Participant characteristics and psychological data.

	Female	Male	Total
*n*	30	59	89
**Age (years)**			
Mean, SD	27.8, 12.0	34.2, 14.0	32.1, 13.6
Range	18–68	20–66	18–68
**Handedness**			
Left/right	5/25	4/55	9/80
**CD-RISC scores**			
Mean, SD	58.4, 16.3	58.3, 15.0	58.3, 15.4
Range	21–95	16–93	16–95

*SD* standard deviation, *CD-RISC* Connor–Davidson Resilience Scale.

### Psychological data

To assess individual psychological resilience, we used the CD-RISC, which is a self-rated questionnaire^31^. Total CD-RISC scores range from 0 to 100, and higher CD-RISC scores indicate greater resilience. The mean and SD of the CD-RISC total score were 58.3 and 15.4 for all participants (*n* = 89); 58.3 and 15.0 for male participants (*n* = 59); and 58.4 and 16.3 for female participants (*n* = 30). These data were similar to data from another study of healthy volunteers^32^. The Shapiro–Wilk test did not reject the hypothesis of the normality of the data distribution for the total CD-RISC scores for all participants (*p* = 0.22).

### Task performance

**Figure 1:**
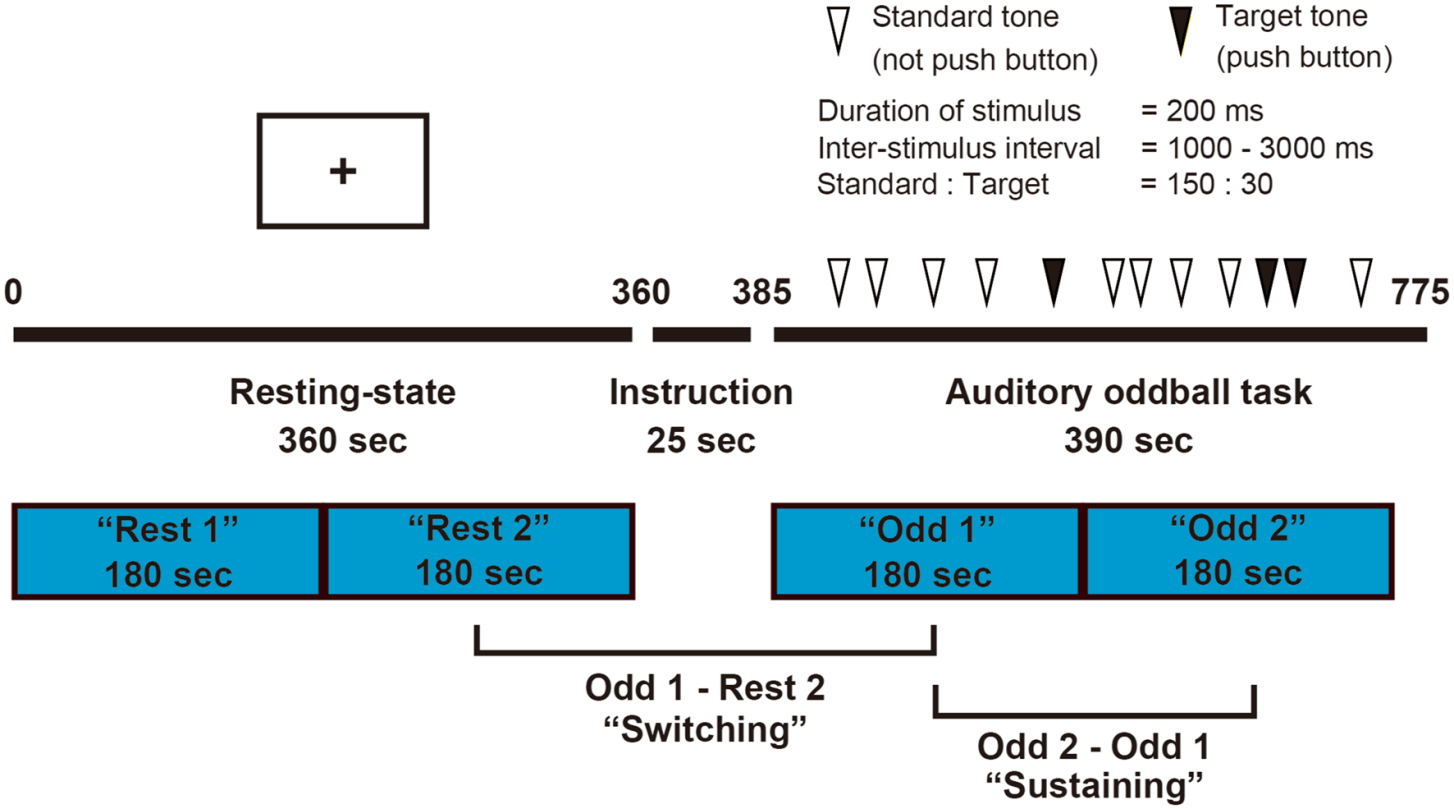
Overview of the time course of functional MRI and time windows for FC analysis. *FC* functional connectivity, *MRI* magnetic resonance imaging. The image was created using Adobe Illustrator software (ver. Creative Cloud 2020).

Behavioural logs during the auditory oddball task were fully completed for 81 of 89 participants. As indices of task performance, we used reaction time (RT), coefficient of variation (CV), commission error rate, and omission error rate. For each participant, the parameters of task performance were calculated separately for the following two time windows (Fig. 1): (1) the 180 s at the beginning of the auditory oddball task [“Odd 1”] and (2) the subsequent 180 s of the task [“Odd 2”]. To equalise the size of the time windows, the last 30 s of the task were not used.

No significant differences were observed between the average RT during “Odd 1” [RT_Odd 1_] and the average RT during “Odd 2” [RT_Odd 2_], between the CV during “Odd 1” [CV_Odd 1_] and the CV during “Odd 2” [CV_Odd 2_], or between the commission error rates and omission error rates in “Odd 1” and “Odd 2” (Table 2a).

**Table 2:** Differences in task performance between the “Odd 1” period and the “Odd 2” period and correlations between CD-RISC scores and task performance.

	“Odd 1”	“Odd 2”	*t*(80)	*p*-value
**a. Differences in task performance between the “Odd 1” and “Odd 2” periods**				
Mean reaction time (ms)	393.4	400.0	1.559	0.12
Coefficient of variation	0.141	0.143	0.173	0.86
Mean commission error rate (%)	0.223	0.258	0.393	0.70
Mean omission error rate (%)	0.794	0.705	− 0.137	0.90

	Spearman's *ρ*	*p*-value		
**b. Correlations between CD-RISC scores and task performance**				
RT_Odd 1_	0.075	0.51		
CV_Odd 1_	0.083	0.46		
RT_Odd 2_	− 0.016	0.89		
CV_Odd 2_	− 0.132	0.24		
RT_Odd 2_ − RT_Odd 1_	− 0.170	0.13		
CV_Odd 2_ − CV_Odd 1_	− 0.205	0.07		
RT_Odd 2_/RT_Odd 1_	− 0.162	0.15		
CV_Odd 2_/CV_Odd 1_	− 0.206	0.07		

*RT* reaction time, *CV* Coefficient of variation, *Spearman's ρ* Spearman’s rank-order correlation coefficient, *CD-RISC* Connor–Davidson Resilience Scale.

The differences and ratios of the RT (RT_Odd 2_ − RT_Odd 1_ and RT_Odd 2_/RT_Odd 1_) and differences and ratios of the CV (CV_Odd 2_ − CV_Odd 1_ and CV_Odd 2_/CV_Odd 1_) were not normally distributed (*p* = 0.0002, 0.0055, 0.0005, and < 0.0001, respectively).

Spearman’s rank correlation analysis showed no correlation between CD-RISC scores and task performance (Table 2b).

We tested the possibility that age and task performance were correlated, and found a weak correlation for RT_Odd 1_ out of eight task performance variables and no correlation between age and the other seven variables (see Supplementary Information 1.1 and Table S1, for details).

To examine the possible effects of age, partial rank correlation coefficients with age as the control variable were calculated, and showed no correlation between CD-RISC scores and task performance (see Supplementary Information 1.2 and Table S2).

Regarding the relationship between CD-RISC score and behaviour, task performance was also compared between the low resilience group (*n* = 41, CD-RISC score ≤ 58) and the high resilience group (*n* = 40, CD-RISC score ≥ 59). We found no significant difference in task performance between the two groups (see Supplementary Information 1.3 and Table S3).

### Functional connectivity

We conducted a region of interest (ROI)-to-ROI FC analysis using CONN^33^. We used ROIs from the Stanford FIND atlas (https://findlab.stanford.edu/research)^20^. This atlas comprises 90 ROIs, including 10 dorsal DMN ROIs and 9 ventral DMN ROIs, and is widely used^34–36^.

For each participant, the preprocessed fMRI time series for all voxels in the 19 ROIs was extracted and averaged separately for the following four time windows (Fig. 1): (1) the first 180 s of the resting state [“Rest 1”], (2) the second 180 s of the resting state [“Rest 2”], (3) the 180 s at the beginning of the auditory oddball task [“Odd 1”], and (4) the subsequent 180 s of the task [“Odd 2”]. To equalise the size of the time windows, the last 30 s of the task were not used. ROI-to-ROI FC was defined as the Fisher-transformed bivariate correlation coefficients for each pair among the 19 regions.

**Figure 2:**
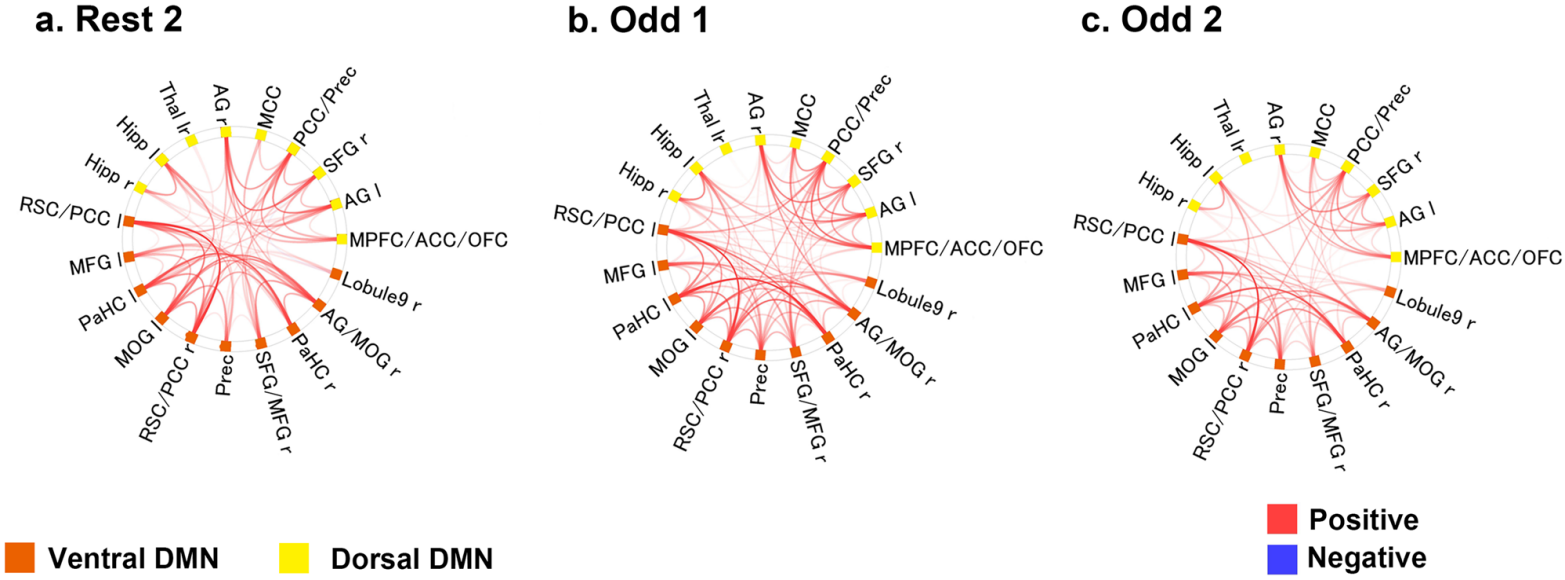
Functional connectivity analyses within the ventral and dorsal DMN. (**a**) FCs of the “Rest 2” period. (**b**) FCs of the “Odd 1” period. (**c**) FCs of the “Odd 2” period. The red line indicates a significant “positive” correlation among the ROIs at both ends. The blue line indicates a significant “negative” correlation among the ROIs at both ends. (FDR-corrected *p* < 0.05). Orange ROIs: ventral DMN. Yellow ROIs: dorsal DMN. *DMN* default mode network, *ROI* region of interest, *FDR* false discovery rate, *r* right, *l* left, *lr* left and right, *Hipp* hippocampus, *Thal* thalamus, *AG* angular gyrus, *MCC* midcingulate cortex, *PCC* posterior cingulate cortex, *Prec* precuneus, *SFG* superior frontal gyrus, *MPFC* medial prefrontal cortex, *ACC* anterior cingulate cortex, *OFC* orbitofrontal cortex, *Lobule 9* lobule 9 of the cerebellum, *MOG* middle occipital gyrus, *PaHC* parahippocampal cortex, *MFG* middle frontal gyrus, *RSC* retrosplenial cortex. The connectome rings were generated in CONN-fMRI Functional Connectivity toolbox (ver. 17f.; https://www.nitrc.org/projects/conn). The whole image was edited in Adobe Illustrator software (ver. Creative Cloud 2020).

Significant positive FCs within the dorsal and ventral DMN were extensively observed at each time window (“Rest 2”, “Odd 1”, and “Odd 2”) (Fig. 2). Almost all the FCs were observed within the ventral DMN or within the dorsal DMN, and some FCs were observed between the ventral DMN and dorsal DMN.

**Figure 3:**
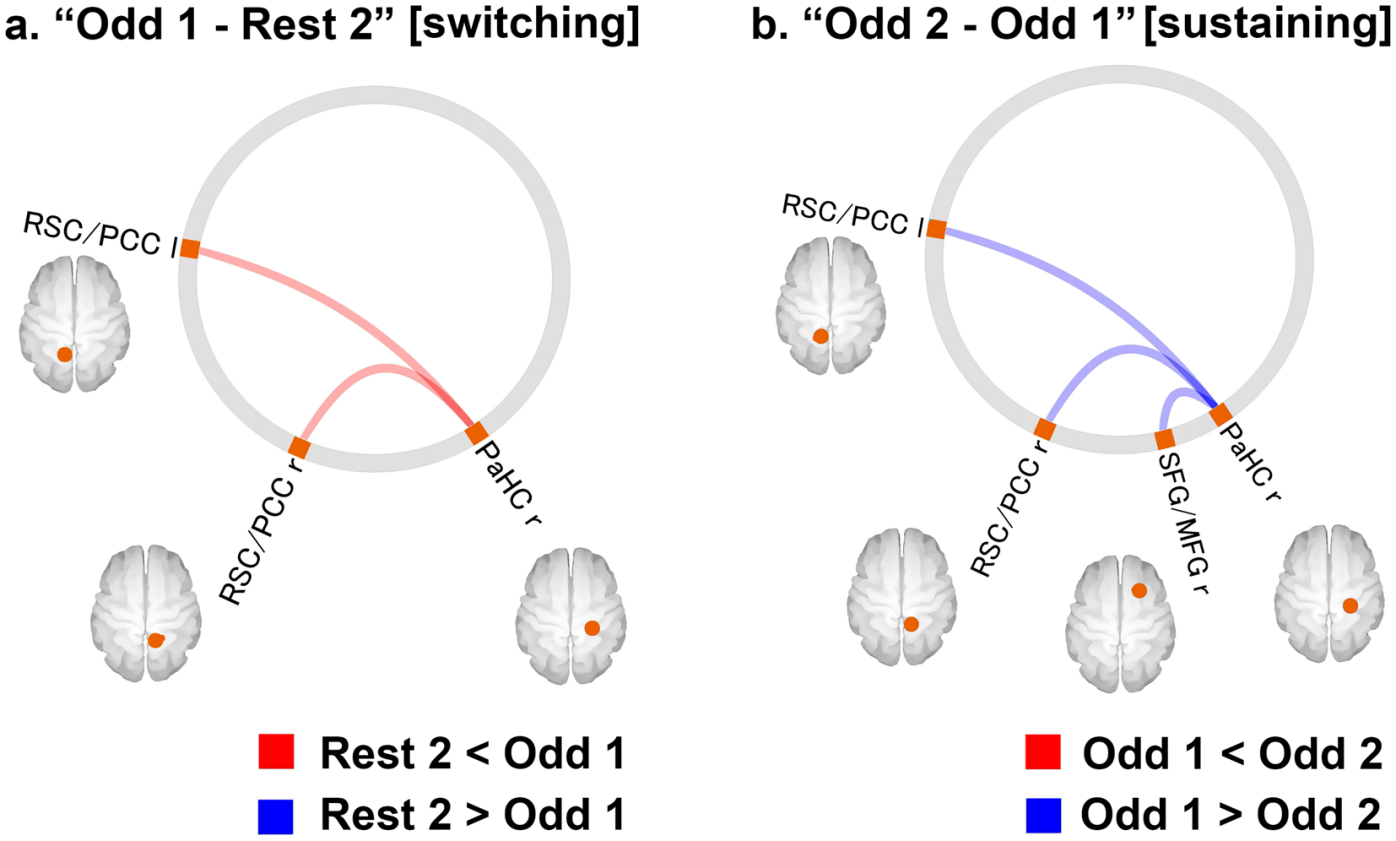
(**a**) FCs with a significant difference between the “Rest 2” and “Odd 1” [switching] periods (FDR-corrected *p* < 0.05). The red line indicates that the FC values in the “Odd 1” period were significantly higher than those in the “Rest 2” period. (**b**) FCs with a significant difference between the “Odd 2” and “Odd 1” [sustaining] periods (FDR-corrected *p* < 0.05). The blue line indicates that the FC values in the “Odd 2” period were significantly lower than those in the “Odd 1” period. Orange ROIs: ventral DMN. Yellow ROIs: dorsal DMN. *FC* functional connectivity, *FDR* false discovery rate, *DMN* default mode network, *r* right, *l* left, *RSC* retrosplenial cortex, *PCC* posterior cingulate cortex, *SFG* superior frontal gyrus, *MFG* middle frontal gyrus, *PaHC* parahippocampal cortex. The connectome rings were generated in CONN-fMRI Functional Connectivity toolbox (ver. 17f.; https://www.nitrc.org/projects/conn). The whole image was edited in Adobe Illustrator software (ver. Creative Cloud 2020).

FCs with significant differences between the “Rest 2” and “Odd 1” [switching] time windows and between the “Odd 1” and “Odd 2” [sustaining] time windows within the DMN are shown in Fig. 3 and Table 3. FC values between the right parahippocampal cortex (PaHC) and bilateral retrosplenial cortex/posterior cingulate cortex (RSC/PCC) were significantly higher in the “Odd 1” window than in the “Rest 2” window (Fig. 3a). FC values between the right PaHC and the bilateral RSC/PCC, as well as those between the right PaHC and the right superior frontal gyrus/middle frontal gyrus (SFG/MFG), were significantly lower in the “Odd 2” window than in the “Odd 1” window (Fig. 3b).

**Table 3:** Comparison of FCs in the DMN between two periods in which FC significantly differed.

ROI-to-ROI analysis unit	T(86)	*p*-unc	*p*-FDR
**a. Between “Rest 2” and “Odd 1” (“Odd 1 − Rest 2”) periods [switching]**			
*RSC/PCC l*			
PaHC r	3.94	0.0002	0.003
*PaHC r*			
RSC/PCC l	3.94	0.0002	0.003
RSC/PCC r	2.98	0.0037	0.0336
**b. Between “Odd 1” and “Odd 2” (“Odd 2 − Odd 1”) periods [sustaining]**			
*PaHC r*			
RSC/PCC r	− 2.84	0.0057	0.0497
SFG/MFG r	− 2.82	0.0059	0.0497
RSC/PCC l	− 2.7	0.0083	0.0497

*FC* functional connectivity, *DMN* default mode network, *ROI* region of interest, *p-unc* *p*-uncorrected, *FDR* false discovery rate, *l* left, *r* right, *RSC/PCC* retrosplenial cortex/posterior cingulate cortex, *PaHC* parahippocampal cortex, *SFG/MFG* superior frontal gyrus/middle frontal gyrus.

**Figure 4:**
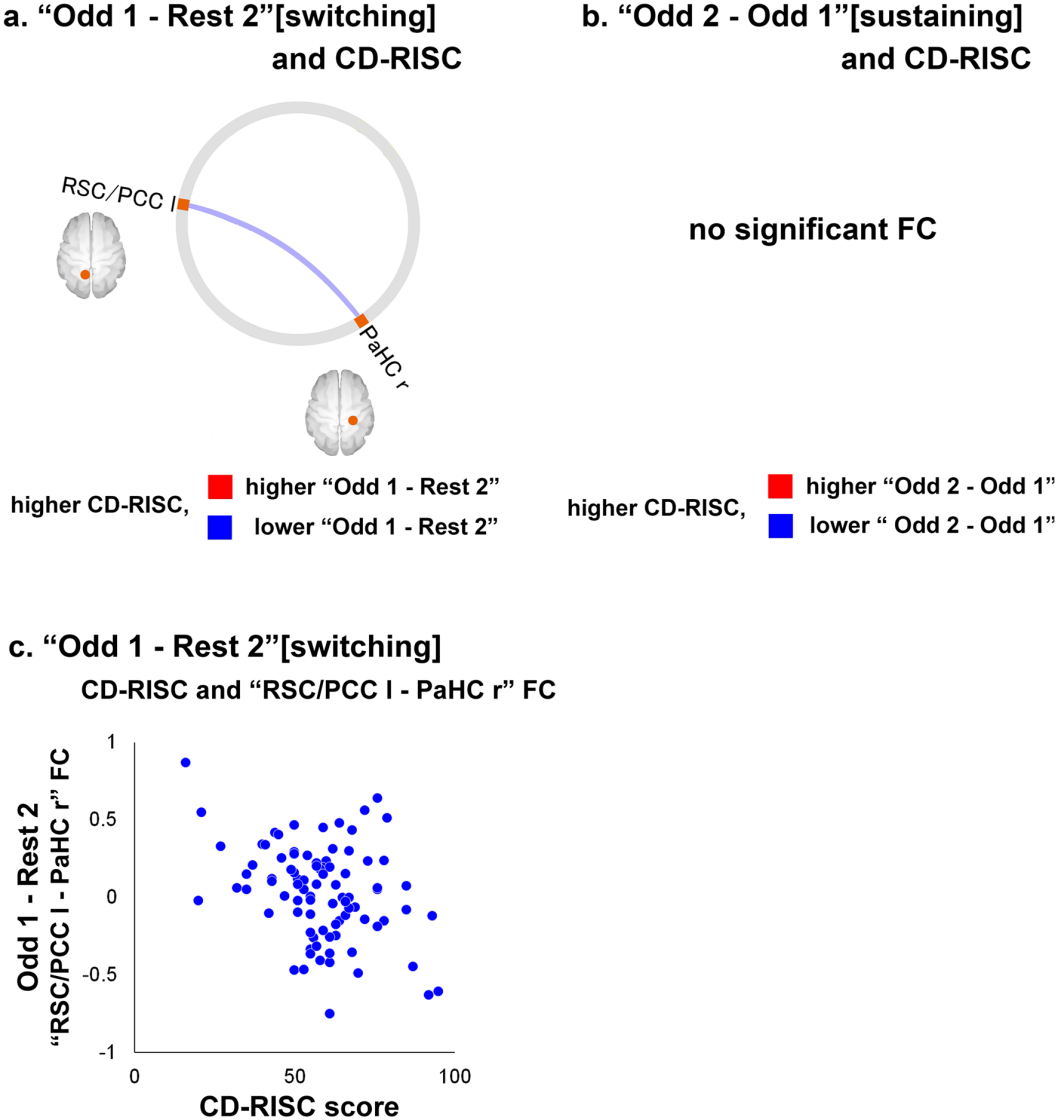
(**a**) FCs with a significant correlation between CD-RISC scores and the difference between the “Rest 2” and “Odd 1” (“Odd 1 − Rest 2” [switching]) periods (FDR-corrected *p* < 0.05). The blue line indicates that there was a negative correlation between the value of the “Odd 1 − Rest 2” period and CD-RISC scores. (**b**) FCs with a significant correlation between CD-RISC scores and the difference between the “Odd 1” and “Odd 2” (“Odd 2 − Odd 1” [sustaining]) periods (FDR-corrected *p* < 0.05). There was no significant FC. (**c**) Scatterplot of the relationship between CD-RISC scores and the “RSC/PCC l-PaHC r” FC of “Odd 1 − Rest 2”. The X-axis of the scatterplot indicates the CD-RISC scores. The Y-axis indicates the “RSC/PCC l-PaHC r” FC values of “Odd 1 − Rest 2” (subtraction of the Fisher’s Z-score of “Rest 2” from that of “Odd 1”). Orange ROIs: ventral DMN. Yellow ROIs: dorsal DMN. *ROI* region of interest, *DMN* default mode network, *r* right, *l* left, *lr* left and right, *PCC* posterior cingulate cortex, *PaHC* parahippocampal cortex, *RSC* retrosplenial cortex, *CD-RISC* Connor–Davidson Resilience Scale, *FC* functional connectivity. The connectome ring was generated in CONN-fMRI Functional Connectivity toolbox (ver. 17f.; https://www.nitrc.org/projects/conn) and the scatter plot was generated in Microsoft Excel 2016. The whole image was edited in Adobe Illustrator software (ver. Creative Cloud 2020).

Correlations between CD-RISC scores and the difference in FC values between the “Rest 2” and “Odd 1” [switching] windows, as well as between the “Odd 1” and “Odd 2” [sustaining] windows within the DMN, are shown in Fig. 4 and Table 4. CD-RISC total scores were negatively correlated with the difference in FC values during the “Odd 1 − Rest 2” [switching] window between the right PaHC and left RSC/PCC (Fig. 4a), and a scatterplot of the relationship between CD-RISC scores and the FC is shown in Fig. 4c. CD-RISC total scores were not significantly correlated with the difference in FC values in the “Odd 2 − Odd 1” [sustaining] window (Fig. 4b). Additionally, when age and sex were not included as covariates, CD-RISC total scores were negatively correlated with the difference in FC values during the “Odd 1 − Rest 2” window between the right PaHC and the left RSC/PCC [T(87) =  − 3.48, uncorrected *p* = 0.0008, false discovery rate (FDR)-corrected *p* = 0.0078]; thus, the results were consistent regardless of whether age and sex were included as covariates. Additional analysis of right-handed participants (*n* = 80) showed that CD-RISC scores were correlated with the difference in FC values during the “Odd 1 − Rest 2” [switching] window between the right PaHC and left RSC/PCC, with age and sex as covariates of an FC analysis [T(76) =  − 2.83, uncorrected *p* = 0.0059, FDR-corrected *p* = 0.05].

**Table 4:** FCs with a significant correlation between total CD-RISC score and the difference in FC between the different periods.

ROI-to-ROI analysis unit	T(85)	*p*-unc	*p*-FDR
**a. Between “Rest 2” and “Odd 1” (“Odd 1 − Rest 2”) periods [switching]**			
*RSC/PCC l*			
PaHC r	− 2.91	0.0047	0.042
**b. Between “Odd 1” and “Odd 2” (“Odd 2 − Odd 1”) periods [sustaining]**			
*No significant FC*			
–	–	–	–

*FC* functional connectivity, *ROI* region of interest, *p-unc* *p*-uncorrected, *FDR* false discovery rate, *l* left, *r* right, *RSC/PCC* retrosplenial cortex/posterior cingulate cortex, *PaHC* parahippocampal cortex, *CD-RISC* Connor–Davidson Resilience Scale.

In this study, we set the DMN as the main network of interest. However, we also investigated the salience network (SN) and the attention network (AN). Regarding the SN, it is known that resilience involves regions such as the anterior cingulate cortex^28^ and the insula^29^. Additionally, the SN has been identified as the switching network between rest and task states^30^, and dysregulation of the equilibrium between the SN and the DMN in subjects with PTSD compared with controls has been reported^37^. Because we used an attention task, we also investigated the AN^27^. As for the DMN FC, the FC value for each time window of “Rest 2”, “Odd 1”, and “Odd 2” was calculated within the SN and the AN. The correlations between “Odd 1 − Rest 2”, “Odd 2 − Odd 1”, and CD-RISC were investigated using age and sex as nuisance covariates. Both within the SN and within the AN, there was no significant correlation (FDR-corrected *p* < 0.05) between CD-RISC score and the FC value difference of both “Odd 1 − Rest 2” and “Odd 2 − Odd 1”. More details of the FC analysis of the SN and the AN are provided in Supplementary Information 1.4.

## Discussion

To our knowledge, this is the first study to investigate the neural underpinnings of resilience in healthy participants, focusing on the association between resilience and the dynamic aspects of FCs within the DMN during “switching” (from the resting state to a task) and “sustaining” (during a task) behaviours. We observed FC changes within the ventral DMN both during “switching” and “sustaining”. Most importantly, FC changes during “switching” were correlated with CD-RISC scores.

Across the whole participant group, we observed an increase in FCs within the DMN (more specifically, FC between the PaHC and the RSC/PCC) during the period from the latter half of the resting state to the beginning period of the task state (“switching”). The DMN was originally identified as a set of brain regions that consistently demonstrated decreased brain activity during a variety of cognitive tasks, compared with baseline^9,10^. Although the DMN has been regarded as comprising regions that are typically deactivated during tasks, DMN activity may persist during both task and rest conditions if the task is not sufficiently challenging^38^. Regarding “connectivity” (not “activity”) within the DMN, the magnitude and direction of the changes were inconsistent^39–42^. This inconsistency in the literature is likely a result of multiple factors, with task demand differences as a primary factor. In studies that used relatively demanding tasks such as a visuoverbal target detection test involving working memory^41^ or a two-back working memory paradigm^40^, smaller FCs within the DMN were found during the tasks compared with the resting state. In contrast, Goparaju et al. used a less demanding visual motion task^42^ and found larger FCs within the DMN during the task compared with the resting state. The authors explain that the task they used was a simple task with a high accuracy rate and was insufficiently difficult to prevent DMN activation^42^. The auditory oddball task that we used in this study was also easy for all participants, and showed a low error rate. Another explanation may be that the DMN regions play a role in the auditory oddball task. One fMRI study that used an auditory target-stimuli detection task, showed that target processing was associated with activation of DMN regions such as the posterior cingulate cortex, middle frontal gyrus, and parahippocampal cortex^43^. These two explanations suggest that DMN regions are not fully suppressed during a task, but either continue to operate or are even actively engaged by the task demands. We also found a decreased FC within the DMN from the beginning period to the following period of the task state (“sustaining”). The time course of brain activity during periods of prolonged cognitive load has not been thoroughly investigated. However, using a continuous psychomotor vigilance test, Gui et al. demonstrated decreased post-test vs. pre-test spontaneous activity in the DMN and interpreted this change in brain activity as a neural underpinning of mental fatigue^44^. Thus, our results are in line with those of Gui et al., although we investigated connectivity instead of activity. In the present study, we found FC attenuation during the task in three connections, two of which involved the PCC. This is interesting, as the PCC has been implicated in the maintenance of vigilance^45^.

Notably, regarding the connectivity within the DMN (i.e., the FC between the right PaHC and the bilateral RSC/PCC), the increase in FC within the DMN during “switching” was negatively correlated with CD-RISC scores. As mentioned above, the FC for the PaHC-RSC/PCC increased during the switching phase and reduced during the sustaining phase across participants. This result may indicate that the increase in FCs within the DMN (more specifically, the FC of PaHC-RSC/PCC) during the switching phase was intensified in participants with low resilience and attenuated in those with high resilience. This suggests that FC in the DMN is more homeostatic in the face of psychological demands in participants with high resilience. Interestingly, Zamoscik et al. reported that the FCs of the PaHC-RSC/PCC during sad mood induction were larger in participants with a diagnosis of depression (remitted) than in controls. Further, in participants diagnosed with depression, those with sadder mood and more rumination in daily life showed the largest FCs^46^. Although the participants in the present study were healthy people, the similarity between our data and the connectivity reported by Zamoscik et al. suggest that over-reactivity of this specific connection is a common neural marker both of less resilient normal participants and of those with depression. Resilience is a protective factor against the development of psychiatric disorders such as PTSD^47^ and depression^48^. Focusing on “changes” in brain connectivity that occur during the transition between rest and task behaviour, rather than measuring changes during rest or a task alone, may capture superior neural markers of resilience as well as vulnerability to psychiatric diseases with stress aetiology.

As this sample had a wide age range, there was a possibility that age would correlate with cognitive task performance^49^ and indirectly affect the results. We therefore investigated the relationship between age and task performance and CD-RISC score. We concluded that the overall contribution of age was probably limited, on the basis of the following results: (1) only one of the eight task performance parameters was weakly correlated with age (Table S1 in Supplementary Information), (2) there were no significant correlations between CD-RISC scores and task performance (Table 2b), (3) there were no significant partial rank correlations between CD-RISC scores and task performance with age as a control variable (Supplementary Table S2), (4) there was no difference in task performance between the low resilience group and the high resilience group (Supplementary Table S3), (5) the negative correlation between CD-RISC scores and the difference in FC values of the right PaHC and the left RSC/PCC during the “Odd 1 − Rest 2” window were consistent regardless of whether age and sex were included as covariates.

This study had several limitations. First, all participants were relatively young. Therefore, the results cannot be generalised to the general population, including elderly people. Second, we used a cross-sectional design. Thus, it is impossible to clarify the causal relationship between resilience and FC changes within the DMN. It should also be noted that the oddball task used here was a relatively easy task. As mentioned above, task difficulty is quite likely to affect results. Furthermore, as there was an “instruction” section for 25 s between “Rest 2” and “Odd 1”, it is possible that “switching” was not measured specifically in this experimental paradigm. To at least partly address this issue, a comparison of the difference in FC values between “Rest 2” and “Odd 1” (“Odd 1 − Rest 2”) and the difference in FC values between “Rest 2” and “Odd 2” (“Odd 2 − Rest 2”) is needed; if the former difference is different from the latter, this “difference of difference” could be interpreted as reflecting the process of “switching” rather than simple reflection of difference between the task and rest. Therefore, we investigated this “difference of difference” [(“Odd 2 − Rest 2”) − (“Odd 1 − Rest 2”)], which is equivalent to (“Odd 2 − Odd 1”) (Fig. 3). The results showed that the FCs of left and right RSC/PCC and right PaHC were greater for “Odd 1” than for “Odd 2”, and the same FCs were greater for “Odd 1” than for “Rest 2”. These results indirectly suggest that the changes observed in the contrast between (“Odd 1 − Rest 2”) are a result of task switching. The application of multiple task conditions with varying task difficulty and task design (e.g., directly connected rest and task) in future research would address some of these unanswered questions.

In conclusion, we investigated the time course of FC within the DMN during the transition from the resting state to the task state. Higher resilience appeared to be associated with greater homeostasis in the DMN. FC changes within the DMN during the transition from the resting to task state may be a biomarker of resilience.

## Materials and methods

### Participants

We recruited 92 healthy volunteers. The inclusion criteria were (1) provision of written consent for research participation and (2) aged 18–70 years at registration. The exclusion criteria were (1) pregnant or puerperal, mental/neurological disorders, disease that affects the central nervous system metabolism, (2) inability to provide written consent, and (3) contraindications of MRI (medical metallic implants, cardiac pacemaker/prosthetic valve, possibility of pregnancy, metal polishing work history, claustrophobia, permanent tattoos/makeup). Two well-trained psychiatrists confirmed that no participant had any psychiatric disorder or severe medical or neurological illness. Estimated intelligence quotients were obtained using the Japanese version of the Adult Reading Test (JART)^50^, and all participants fell within the normal range. Handedness was determined using the Edinburgh Handedness Inventory. Following the original scoring^51^, participants with a positive score were classified as right-handed. After the experimental procedures were fully explained, all participants provided written informed consent before study participation. The study was approved by the ethics committee of the Kyoto University Graduate School and Faculty of Medicine, and conducted in accordance with the guidelines of the Declaration of Helsinki.

### Psychological questionnaire

#### Connor–Davidson Resilience Scale (CD-RISC)^31^

The CD-RISC is a self-rating questionnaire that assesses an individual’s degree of resilience. It contains 25 items, and responses are on a 5-point Likert-type scale. Total CD-RISC scores range from 0 to 100. Higher CD-RISC scores indicate greater resilience. The original CD-RISC has been validated^31^.

We used the Japanese version of the CD-RISC. The reliability and validity of the Japanese version of the CD-RISC has been examined and confirmed: Cronbach’s alpha coefficient (α = 0.90) and the test–retest correlation (*r* = 0.83, *p* < 0.01) were sufficiently high to confirm reliability. CD-RISC scores are positively associated with the concepts of hardiness, sense of coherence, and social support (*r* = 0.68, 0.50, 0.23, *p* < 0.01, respectively) and negatively associated with perceived stress and Kessler Psychological Distress Scale (K6) scores (*r* =  − 0.54, − 0.44, *p* < 0.01). Hierarchical multiple regression analyses have also confirmed incremental validity of CD-RISC in predicting perceived stress^32^.

### MRI acquisition

MRI acquisition was performed using a 3-T MRI unit (Tim-Trio; Siemens, Erlangen, Germany) with a 40-mT/m gradient and a receiver-only 32-channel phased-array head coil. A 360-s resting-state fMRI scan was acquired using a single-shot gradient-echo echo planar imaging (EPI) pulse sequence. Participants were instructed to visually concentrate on a fixation cross in the centre of the screen and to avoid thinking about anything specific during resting-state data acquisition. Next, they received a 25-s explanation of how to complete the auditory oddball task, and then performed the task for 390 s. The task consisted of 30 pink-noise sounds as target stimuli and 150 pure 400-Hz tones as non-target stimuli (Fig. 1)^52^. Target and non-target stimuli were arranged in a randomised order. All stimuli were presented for 200 ms with a jittered and randomised inter-stimulus interval of 1–3 s in 100-ms units^52^. During the task, participants were instructed to differentiate between target and non-target stimuli by pressing a button with right thumb as fast and accurately as possible after the target stimulus presentation. We measured the RT for all responses to the target stimuli. All participants practiced before entering the scanner and we confirmed that they understood the procedure and were able to perform the 16-s practice session with 100% accuracy. The total fMRI acquisition time was 775 s (Fig. 1). Head movement was minimised within the head coil using foam rubber pads. A dual-echo gradient-echo dataset for B0-field mapping was also acquired for distortion correction. T1-weighted three-dimensional structural images were also acquired using magnetisation-prepared rapid gradient-echo (MPRAGE) sequences.

Additional details are provided in Supplementary Information 2.1.

### Image preprocessing

We corrected the fMRI dataset for EPI distortion using FMRIB’s Utility for Geometrically Unwarping EPIs (FUGUE), which is part of the FSL package (FMRIB’s software library ver. 5.0.9; https://www.fmrib.ox.ac.uk/fsl), using fieldmap data. We removed artefact components and motion-related fluctuations from the images using FMRIB’s ICA-based X-noiseifier (FIX)^53^.

We then processed the preprocessed fMRI and structural MRI data using the CONN-fMRI Functional Connectivity toolbox^33^ (ver. 17f.; https://www.nitrc.org/projects/conn) with the statistical parametric mapping software package SPM12 (Wellcome Trust Centre for Neuroimaging; https://www.fil.ion.ucl.ac.uk/spm).

Before running FIX, we evaluated movement that occurred during fMRI scanning using frame-wise displacement, which quantifies head motion between each volume of functional data^54^. We applied two exclusion criteria: (1) when the number of volumes in which head position was 0.5 mm different from adjacent volumes was more than 25%^55^ and (2) when the maximum head motion was more than 3.0 mm and 3.0°^56^. No participants were excluded under (1), whereas three participants were excluded under (2). Finally, 89 of the 92 participants were included in the FC analysis.

Additional details are provided in Supplementary Information 2.2.

### Functional connectivity analysis

We conducted an ROI-to-ROI FC analysis. We used ROIs from the Stanford FIND atlas (https://findlab.stanford.edu/research). A total of 90 ROIs of resting-state networks have been identified using an independent component analysis of data from healthy individuals^20^. These have been grouped into several networks, including the dorsal and ventral DMN, and are widely used^34–36^. The ventral DMN includes the cerebellar region, which contributes to parallel cortico-cerebellar loops involved in episodic memory/self-reflection^57^. The 10 ROIs in the dorsal DMN were located in the medial prefrontal cortex/anterior cingulate cortex/orbitofrontal cortex (MPFC/ACC/OFC), left angular gyrus (AG l), right superior frontal gyrus (SFG r), posterior cingulate cortex/precuneus (PCC/Prec), midcingulate cortex (MCC), right angular gyrus (AG r), left and right thalamus (Thal lr), left hippocampus (Hipp l), and right hippocampus (Hipp r). The nine ROIs in the ventral DMN were located in the left retrosplenial cortex/posterior cingulate cortex (RSC/PCC l), left middle frontal gyrus (MFG l), left parahippocampal cortex (PaHC l), left middle occipital gyrus (MOG l), right retrosplenial cortex/posterior cingulate cortex (RSC/PCC r), precuneus (Prec), right superior frontal gyrus/middle frontal gyrus (SFG/MFG r), right parahippocampal gyrus (PaHC r), right angular gyrus/middle occipital gyrus (AG/MOG r), and the right cerebellar lobule IX (Lobule9 r).

For each participant, we extracted the preprocessed fMRI time series of all voxels in the 19 ROIs and separately averaged them for the following four time windows (Fig. 1): (1) the first 180 s of the resting state [“Rest 1”], (2) the second 180 s of the resting state [“Rest 2”], (3) the 180 s at the beginning of the auditory oddball task [“Odd 1”], and (4) the subsequent 180 s of the task [“Odd 2”]. To equalise the size of the time windows, the last 30 s of the task were not used. We defined the ROI-to-ROI FC as the Fisher-transformed bivariate correlation coefficients for each pair among the 19 regions, and constructed a 19 × 19 correlation matrix (171 FCs) for each participant for each time window.

### Task performance

To assess differences in task performance between the “Odd 1” and “Odd 2” periods, we calculated the average RT during “Odd 1” (RT_Odd 1_) and “Odd 2” (RT_Odd 2_). The CV was calculated by dividing the standard deviation by the mean. We calculated the average coefficient of variation during the “Odd 1” (CV_Odd 1_) and “Odd 2” (CV_Odd 2_) periods. The commission error rate was calculated by dividing the number of commission errors (button presses for non-target stimuli) by the total number of non-target stimuli. The omission error rate was calculated by dividing the number of omission errors (absent button pushes for target stimuli) by the total number of target stimuli.

### Statistical analysis

Two-tailed *t*-tests were used for comparisons of the average RT, CV, commission error rate, and omission error rate between the “Odd 1” and “Odd 2” periods. To investigate the relationship between differences in task performance and CD-RISC scores, we conducted correlation analyses for the total CD-RISC score and RT_Odd 1_, CV_Odd 1_, RT_Odd 2_, CV_Odd 2_, RT_Odd 2_ − RT_Odd 1_, CV_Odd 2_ − CV_Odd 1_, RT_Odd 2_/RT_Odd 1_, and CV_Odd 2_/CV_Odd 1._ A one-sample Shapiro–Wilk test indicated that the data had a mixed distribution. Therefore, to test the correlations mentioned above, Pearson’s correlation coefficients were used if an initial exploration of the dataset indicated normal distribution of the data, and Spearman’s rank-order correlation coefficients were used if the data were not normally distributed. The demographic and behaviour data analysis were conducted using IBM SPSS Statistics 24.

FC values for the “Rest 2”, “Odd 1”, and “Odd 2” periods within the ventral and dorsal DMN were calculated using CONN FC analysis. We focused on the differences in FC values between the “Rest 2” and “Odd 1” (“Odd 1 − Rest 2” [switching]) periods, and between the “Odd 1” and “Odd 2” (“Odd 2 − Odd 1” [sustaining]) periods. The differences were calculated with age and sex as covariates of an FC analysis. Furthermore, correlations between the total CD-RISC scores and the significant differences between FC values in the “Odd 1 − Rest 2” [switching] and “Odd 2 − Odd 1” [sustaining] periods were also calculated with age and sex as covariates of an FC analysis. The threshold for significance was the FDR-adjusted *p*-value of < 0.05 (two-tailed).

## Supplementary information

Supplementary Information.

## Data Availability

The datasets generated during and/or analysed during the current study are available from the corresponding author on reasonable request.
