# Supplementary material for: Psychological resilience is correlated with dynamic changes in functional connectivity within the default mode network during a cognitive task
Source: Sci Rep. 2020 Oct 20;10:17760. doi: 10.1038/s41598-020-74283-7 (PMC7576164; doi:10.1038/s41598-020-74283-7)
Supplement: Supplementary file 1 — Supplementary Information. [file 41598_2020_74283_MOESM1_ESM.docx]

**Supplementary Information**

**Psychological resilience is correlated with dynamic changes in functional connectivity within the default mode network during a cognitive task**

Takashi Miyagi^1^, Naoya Oishi^2*^, Kei Kobayashi^1^, Tsukasa Ueno^1,3^, Sayaka Yoshimura^4^, Toshiya Murai^1^, and Hironobu Fujiwara^1,5^

^1^ Department of Psychiatry, Graduate School of Medicine, Kyoto University, Kyoto, Japan

^2^ Medical Innovation Center, Kyoto University Graduate School of Medicine, Kyoto, Japan

^3^ Integrated Clinical Education Center, Kyoto University Hospital, Kyoto, Japan

^4^ Department of Neurodevelopmental Psychiatry, Habilitation and Rehabilitation, Kyoto University, Kyoto, Japan

^5^ Artificial Intelligence Ethics and Society Team, RIKEN Center for Advanced Intelligence Project, Tokyo, Japan

*noishi@kuhp.kyoto-u.ac.jp

**1. Supplementary RESULTS**

**1.1 Correlations between age and task performance**

We examined the correlations between age and task performance using Spearman’s correlation coefficient. There was a weak correlation only of the mean reaction time (RT) for “Odd 1” period (RT_Odd 1_) out of eight task performance variables and no correlation between age and the other seven variables (Supplementary Table S1).

**1.2 Correlations between Connor–Davidson Resilience Scale (CD-RISC) scores and task performance with age as a control variable**

Partial rank correlation coefficients between CD-RISC scores and task performance were calculated with age as a control variable. No correlation was found between CD-RISC scores and task performance (Supplementary Table S2).

**1.3 Comparison of task performance between low resilience group and high resilience group**

All participants were divided into a low resilience group and a high resilience group using the median CD-RISC score for all participants. Then we compared task performance between the two groups using the Wilcoxon rank-sum test. The median CD-RISC score for all participants with full task performance log (*n* = 81) was 58. Forty-one participants whose CD-RISC score was ≤58 were assigned to a low resilience group and 40 participants whose CD-RISC score was ≥59 were assigned to a high resilience group. Group comparisons showed no significant difference between the two groups (Supplementary Table S3).

**1.4 Functional connectivity (FC) analysis of the salience network (SN) and the attention network (AN)**

Regarding the SN, it is known that resilience involves regions such as the anterior cingulate cortex^[1]^ and the insula^[2]^. Additionally, the SN has been shown to be the switching network between rest and task states^[3]^, and dysregulation of the equilibrium between the SN and the default mode network (DMN) in subjects with posttraumatic stress disorder compared with controls has been reported^[4]^. Because we used an attention task, we also investigated the AN^[5]^.

To conduct a region of interest (ROI)-to-ROI analysis of the SN, we used the ROIs from the Stanford FIND atlas (http://findlab.stanford.edu/research). ROIs have been identified as resting-state networks using an independent component analysis of data from healthy individuals^[6]^. This atlas includes 90 ROIs, grouped into several networks, including the SN as well as the DMN, and has been widely used by previous studies^[7,8]^. The SN includes the anterior SN and the posterior SN, and 19 ROIs were located in the left middle frontal gyrus, left insula, anterior cingulate cortex/medial prefrontal cortex/supplementary motor area, right middle frontal gyrus, right insula, left lobule VI/crus I, right lobule VI/crus I, left middle frontal gyrus, left supramarginal gyrus/inferior parietal gyrus, left precuneus, right midcingulate cortex, right superior parietal gyrus/precuneus, right supramarginal gyrus/inferior parietal gyrus, left thalamus, left lobule VI, left posterior insula/putamen, right thalamus, right lobule VI, and right posterior insula.

The AN includes the dorsal AN and the ventral AN. The 12 ROIs of the dorsal AN and the 12 ROIs of the ventral AN are spherical clusters, each of which has a 10-mm diameter; the cluster peak coordinates have been demonstrated in a previous functional magnetic resonance imaging (fMRI) study^[9]^. The dorsal and the ventral AN ROIs were located in the left and right superior frontal gyrus, left and right superior parietal lobule, left and right superior parietal lobule, left and right inferior temporal gyrus, left and right precuneus, left and right precentral gyrus, left and right middle frontal gyrus, left and right inferior parietal gyrus, left and right middle temporal gyrus, left and right cingulate gyrus, left and right cingulate sulcus, and left and right insula.

We analysed FC within the SN and within the AN in the same way as the main analysis of the DMN. For each participant, we extracted the preprocessed fMRI time series of all voxels in all ROIs of each network and separately averaged them for the following four time windows (Figure 1): 1) the first 180 s of the resting state [“Rest 1”], 2) the second 180 s of the resting state [“Rest 2”], 3) the 180 s at the beginning of the auditory oddball task [“Odd 1”], and 4) the subsequent 180 s of the task [“Odd 2”]. To equalise the size of the time windows, the last 30 s of the task were not used. We defined the ROI-to-ROI FC as the Fisher-transformed bivariate correlation coefficients for each pair among the regions of each network, and constructed a correlation matrix for each participant for each time window.

FC values for the “Rest 2”, “Odd 1”, and “Odd 2” periods were calculated using the CONN FC analysis separately for the SN and the AN. As with the main analysis, we focused on the differences in FC values between the “Rest 2” and “Odd 1” (“Odd 1 − Rest 2” [switching]) periods, and between the “Odd 1” and “Odd 2” (“Odd 2 − Odd 1” [sustaining]) periods. The differences were calculated with age and sex as covariates of an FC analysis. Furthermore, correlations between the total CD-RISC scores and the significant differences between FC values in the “Odd 1 − Rest 2” [switching] and “Odd 2 − Odd 1” [sustaining] periods were also calculated with age and sex as covariates of an FC analysis. The threshold for significance was a false discovery rate (FDR)-corrected *p*-value of <0.05 (two-tailed).

Both within the SN and within the AN, there was no significant correlation between CD-RISC scores and the FC value difference of both “Odd 1 − Rest 2” and “Odd 2 − Odd 1”.

**2. Supplementary METHODS**

**2.1. MRI Acquisition**

MRI acquisition was performed using a 3-Tesla MRI unit (Tim-Trio; Siemens, Erlangen, Germany) with a 40-mT/m gradient and a receiver-only 32-channel phased-array head coil. A 360-s resting-state fMRI scan was acquired using a single-shot gradient-echo echo planar imaging (EPI) pulse sequence with the following parameters: echo time (TE), 30 ms; repetition time (TR), 2500 ms; flip angle, 80°; field of view (FOV), 212 × 212 mm; matrix size, 64 × 64; in-plane spatial resolution, 3.3125 × 3.3125 mm^2^; 40 total axial slices; and slice thickness, 3.2 mm with 0.8-mm gaps in ascending order. Participants were instructed to visually concentrate on a fixation cross in the centre of the screen and to avoid thinking about anything specific during resting-state data acquisition. Next, they received a 25-s explanation about how to complete the auditory oddball task, and then performed the task for 390 s. The task consisted of 30 pink-noise sounds as target stimuli and 150 pure 400-Hz tones as non-target stimuli (Figure 1)^[10]^. Target and non-target stimuli were arranged in a randomised order. The sounds were generated using Audacity 2.1.1 software (https://www.audacityteam.org/). All stimuli were presented using E-prime 2.0 software (Psylab, USA) for 200 ms with a jittered and randomised inter-stimulus interval of 1–3 s in 100-ms units^[10]^. During the task, participants were instructed to differentiate between target and non-target stimuli by pressing a button with right thumb as fast and accurately as possible after the target stimulus presentation. We measured the RT for all responses to target stimuli. All participants practiced before entering the scanner and we confirmed that they understood the procedure and were able to perform the 16-s practice session with 100% accuracy. The total acquisition time for the fMRI was 775 s (Figure 1). Head movement was minimised within the head coil using foam rubber pads. A dual-echo gradient-echo dataset for B0-field mapping was also acquired for distortion correction.

T1-weighted three-dimensional structural images were also acquired using magnetisation-prepared rapid gradient-echo (MPRAGE) sequences with the following parameters: TE, 3.4 ms; TR, 2000 ms; inversion time, 990 ms; FOV, 225 × 240 mm; matrix size, 240 × 256; resolution, 0.9375 × 0.9375 × 1.0 mm^3^; and 208 total axial sections without intersection gaps.

**2.2 Image Preprocessing**

We corrected the fMRI dataset for EPI distortion using FMRIB’s Utility for Geometrically Unwarping EPIs (FUGUE), which is part of the FSL package (FMRIB’s software library ver. 5.0.9; http://www.fmrib.ox.ac.uk/fsl), using fieldmap data. We removed artefact components and motion-related fluctuations from the images using FMRIB’s ICA-based X-noiseifier (FIX)^[11]^.

We then processed the preprocessed fMRI and structural MRI data using the CONN-fMRI Functional Connectivity toolbox^[12]^ (ver. 17f; www.nitrc.org/projects/conn) with the statistical parametric mapping software package SPM12 (Wellcome Trust Centre for Neuroimaging; http://www.fil.ion.ucl.ac.uk/spm). First, we conducted the following processing for all functional images with a default preprocessing pipeline for volume-based analysis. The images were realigned and unwarped, slice-timing corrected, coregistered with structural data, spatially normalised into standard MNI space (Montreal Neurological Institute, Canada), outlier detected (Artifact Detection Tools-based scrubbing [ART; www.nitrc.org/projects/artifact_detect/]), and smoothed using a Gaussian kernel with a full-width-at-half maximum of 8 mm. Structural data were segmented into grey matter, white matter (WM), and cerebrospinal fluid (CSF), and normalised using the same default preprocessing pipeline. Using covariate regression analysis, the principal components of signals from WM and CSF, as well as translational and rotational movement parameters (with another six parameters representing their first-order temporal derivatives), were removed. The effects of nuisance covariates, including fluctuations in fMRI signals from WM, CSF, and their derivatives, as well as realignment parameter noise, were reduced using the implemented CompCor strategy^[13]^. We performed band-pass filtering with a frequency window of 0.01–0.1 Hz, following a previous study^[14]^.

Before running FIX, we evaluated movement that occurred during fMRI scanning using frame-wise displacement, which quantifies head motion between each volume of functional data^[15]^. We applied two exclusion criteria: (1) when the number of volumes in which head position was 0.5 mm different from adjacent volumes was more than 25%^[16]^ and (2) when the maximum head motion was more than 3.0 mm and 3.0 degrees^[17]^. No participants were excluded under (1), whereas three participants were excluded under (2). Finally, 89 of the 92 participants were included in the FC analysis.

**References**

1 Kong, F., Wang, X., Hu, S. & Liu, J. Neural correlates of psychological resilience and their relation to life satisfaction in a sample of healthy young adults. *NeuroImage* **123,** 165–172. https://doi:10.1016/j.neuroimage.2015.08.020 (2015).

2 Waugh, C. E., Wager, T. D., Fredrickson, B. L., Noll, D. C. & Taylor, S. F. The neural correlates of trait resilience when anticipating and recovering from threat. *Soc. Cogn. Affect. Neurosci.* **3,** 322–332. https://doi:10.1093/scan/nsn024 (2008).

3 Sidlauskaite, J. *et al.* Anticipatory processes in brain state switching - evidence from a novel cued-switching task implicating default mode and salience networks. *NeuroImage* **98,** 359–365. https://doi:10.1016/j.neuroimage.2014.05.010 (2014).

4 Sripada, R. K. *et al.* Neural dysregulation in posttraumatic stress disorder: evidence for disrupted equilibrium between salience and default mode brain networks. *Psychosom. Med.* **74,** 904–911. https://doi:10.1097/PSY.0b013e318273bf33 (2012).

5 Kim, H. Involvement of the dorsal and ventral attention networks in oddball stimulus processing: a meta-analysis. *Hum. Brain Mapp.* **35,** 2265–2284. https://doi:10.1002/hbm.22326 (2014).

6 Shirer, W. R., Ryali, S., Rykhlevskaia, E., Menon, V. & Greicius, M. D. Decoding subject-driven cognitive states with whole-brain connectivity patterns. *Cereb. Cortex* **22,** 158–165. https://doi:10.1093/cercor/bhr099 (2012).

7 Figley, C. R., Asem, J. S., Levenbaum, E. L. & Courtney, S. M. Effects of body mass index and body fat percent on default mode, executive control, and salience network structure and function. *Front. Neurosci.* **10,** 234. https://doi:10.3389/fnins.2016.00234 (2016).

8 Smith, R. *et al.* Resting state functional connectivity correlates of emotional awareness. *NeuroImage* **159,** 99–106. https://doi:10.1016/j.neuroimage.2017.07.044 (2017).

9 Yeo, B. T. *et al.* The organization of the human cerebral cortex estimated by intrinsic functional connectivity. *J. Neurophysiol.* **106,** 1125–1165. https://doi:10.1152/jn.00338.2011 (2011).

10 Fujiwara, H. *et al.* Martial arts "Kendo" and the motivation network during attention processing: an fMRI study. *Front. Hum. Neurosci.* **13,** 170. https://doi:10.3389/fnhum.2019.00170 (2019).

11 Griffanti, L. *et al.* ICA-based artefact removal and accelerated fMRI acquisition for improved resting state network imaging. *NeuroImage* **95,** 232–247. https://doi:10.1016/j.neuroimage.2014.03.034 (2014).

12 Whitfield-Gabrieli, S. & Nieto-Castanon, A. Conn: a functional connectivity toolbox for correlated and anticorrelated brain networks. *Brain Connect.* **2,** 125–141. https://doi:10.1089/brain.2012.0073 (2012).

13 Behzadi, Y., Restom, K., Liau, J. & Liu, T. T. A component based noise correction method (CompCor) for BOLD and perfusion based fMRI. *NeuroImage* **37,** 90–101. https://doi:10.1016/j.neuroimage.2007.04.042 (2007).

14 Sun, Y. *et al.* The effects of a mid-task break on the brain connectome in healthy participants: a resting-state functional MRI study. *NeuroImage* **152,** 19–30. https://doi:10.1016/j.neuroimage.2017.02.084 (2017).

15 Power, J. D., Barnes, K. A., Snyder, A. Z., Schlaggar, B. L. & Petersen, S. E. Spurious but systematic correlations in functional connectivity MRI networks arise from subject motion. *NeuroImage* **59,** 2142–2154. https://doi:10.1016/j.neuroimage.2011.10.018 (2012).

16 Nilsonne, G. *et al.* Intrinsic brain connectivity after partial sleep deprivation in young and older adults: results from the Stockholm Sleepy Brain study. *Sci. Rep.* **7,** 9422. https://doi:10.1038/s41598-017-09744-7 (2017).

17 Zhang, J. T. *et al.* Decreased functional connectivity between ventral tegmental area and nucleus accumbens in Internet gaming disorder: evidence from resting state functional magnetic resonance imaging. *Behav. Brain Funct.* **11,** 37. https://doi:10.1186/s12993-015-0082-8 (2015).

| **Supplementary Table S1** | | |
| --- | --- | --- |
| **Correlations between age and task performance** | | |
| Variable | Spearman’s *ρ* | *p*-value |
| RT_Odd 1_ | 0.272 | 0.01 |
| CV_odd 1_ | −0.015 | 0.89 |
| RT_odd 2_ | 0.154 | 0.17 |
| CV_odd 2_ | −0.172 | 0.12 |
| RT_odd 2_ − RT_odd 1_ | −0.101 | 0.37 |
| CV_odd 2_ − CV_odd 1_ | −0.129 | 0.25 |
| RT_odd 2_/RT_odd 1_ | −0.089 | 0.43 |
| CV_odd 2_/CV_odd 1_ | −0.110 | 0.33 |
| Abbreviations: RT, reaction time; CV, coefficient of variation; Spearman's *ρ*, Spearman's rank correlation coefficient | | |

| **Supplementary Table S2** | | | |
| --- | --- | --- | --- |
| **Correlations between task performance and CD-RISC scores with age as control variable** | | | |
| Variable | df | Partial rank  correlation coefficient | *p*-value |
| RT_Odd 1_ | 78 | 0.035 | 0.76 |
| CV_Odd 1_ | 78 | 0.090 | 0.43 |
| RT_Odd 2_ | 78 | −0.037 | 0.74 |
| CV_Odd 2_ | 78 | −0.101 | 0.38 |
| RT_Odd 2_ − RT_Odd 1_ | 78 | −0.152 | 0.18 |
| CV_Odd 2_ − CV_Odd 1_ | 78 | −0.189 | 0.09 |
| RT_Odd 2_/RT_Odd 1_ | 78 | −0.147 | 0.19 |
| CV_Odd 2_/CV_Odd 1_ | 78 | −0.192 | 0.09 |

Abbreviations: RT, reaction time; CV, coefficient of variation; df, degrees of freedom; CD-RISC, Connor–Davidson Resilience Scale

| **Supplementary Table S3** | | | | | |  |
| --- | --- | --- | --- | --- | --- | --- |
| **Comparison of task performance between low resilience group and high resilience group** | | | | | |  |
|  | Low resilience (*n* = 41) | | High resilience (*n* = 40) | |  |  |
|  | CD-RISC score ≤58 | | CD-RISC score ≥59 | |  |  |
| Variable | Mean | SD | Mean | SD | *p*-value |  |
| RT_Odd 1_ (ms) | 391 | 57 | 396 | 61 | 0.73 |  |
| CV_Odd 1_ | 0.14 | 0.08 | 0.14 | 0.07 | 0.68 |  |
| RT_Odd 2_ (ms) | 406 | 90 | 394 | 68 | 0.78 |  |
| CV_Odd 2_ | 0.16 | 0.11 | 0.12 | 0.06 | 0.18 |  |
| RT_Odd 2_ − RT_Odd 1_ (ms) | 14 | 40 | −2 | 34 | 0.13 |  |
| CV_Odd 2_ − CV_Odd 1_ | 0.02 | 0.08 | −0.02 | 0.08 | 0.08 |  |
| RT_Odd 2_/RT_Odd 1_ | 1.03 | 0.09 | 1.00 | 0.08 | 0.14 |  |
| CV_Odd 2_/CV_Odd 1_ | 1.22 | 0.64 | 0.98 | 0.49 | 0.07 |  |
| Abbreviations: RT, reaction time; CV, coefficient of variation; SD, standard deviation;  CD-RISC, Connor–Davidson Resilience Scale | | | | | | |
